# The design of a low literacy decision aid about rheumatoid arthritis medications developed in three languages for use during the clinical encounter

**DOI:** 10.1186/s12911-014-0104-8

**Published:** 2014-11-25

**Authors:** Jennifer L Barton, Christopher J Koenig, Gina Evans-Young, Laura Trupin, Jennie Anderson, Dana Ragouzeos, Maggie Breslin, Timothy Morse, Dean Schillinger, Victor M Montori, Edward H Yelin

**Affiliations:** Department of Medicine, University of California, San Francisco, CA USA; Division of Hospital & Specialty Medicine, Portland Veterans Affairs Medical Center, 3710 SW US Veterans Hospital Road, Portland, OR 97239 USA; Phillip R. Lee Institute for Health Policy Studies, San Francisco, CA USA; San Francisco, CA USA; Brooklyn, NY USA; Knowledge and Evaluation Research Unit, Mayo Clinic, Rochester, MN 55905 USA

**Keywords:** Decision aid, Rheumatoid arthritis, Health literacy

## Abstract

**Background:**

Shared decision-making in rheumatoid arthritis (RA) care is a priority among policy makers, clinicians and patients both nationally and internationally. Demands on patients to have basic knowledge of RA, treatment options, and details of risk and benefit when making medication decisions with clinicians can be overwhelming, especially for those with limited literacy or limited English language proficiency. The objective of this study is to describe the development of a medication choice decision aid for patients with rheumatoid arthritis (RA) in three languages using low literacy principles.

**Methods:**

Based on the development of a diabetes decision aid, the RA decision aid (RA Choice) was developed through a collaborative process involving patients, clinicians, designers, decision-aid and health literacy experts. A combination of evidence synthesis and direct observation of clinician-patient interactions generated content and guided an iterative process of prototype development.

**Results:**

Three iterations of RA Choice were developed and field-tested before completion. The final tool organized data using icons and plain language for 12 RA medications across 5 issues: frequency of administration, time to onset, cost, side effects, and special considerations. The tool successfully created a conversation between clinician and patient, and garnered high acceptability from clinicians.

**Conclusions:**

The process of collaboratively developing an RA decision aid designed to promote shared decision making resulted in a graphically-enhanced, low literacy tool. The use of RA Choice in the clinical encounter has the potential to enhance communication for RA patients, including those with limited health literacy and limited English language proficiency.

## Background

Significant advances have been made in treatment options and strategies for rheumatoid arthritis (RA), one of the most common forms of inflammatory arthritis. These advances have shifted the goal of therapy from symptom relief to sustained remission. Over the past twenty years, 10 new disease-modifying anti-rheumatic drugs (DMARDs) have been approved by the FDA for use in RA. These innovations have improved outcomes, but introduced high complexity for patients and clinicians when making treatment decisions about individual agents and their use in combination. Like the treatment of many other chronic diseases, adherence to biologic DMARDs can be poor with persistence rates ranging from 42-71% [1].

Despite advances in therapy, variation in outcomes persists, with certain populations experiencing poorer outcomes [2,3]. In particular, RA patients who are non-white, immigrants, or who have limited English language proficiency have been found to have higher disease activity and poorer function despite access to effective therapies [4]. In addition, limited health literacy has also been associated with greater disability [5]. We define health literacy using the Affordable Care Act of 2010 definition: “the degree to which an individual has the capacity to obtain, communicate, process, and understand basic health information and services to make appropriate health decisions”. One potential mechanism for persistent variation in outcomes is variation in the interpersonal processes of care, such as patient-provider communication. We have found that nearly one-third of adults with RA report suboptimal shared decision-making (SDM) communication. Patients with lower education, limited health literacy, lower trust in physician and limited English language proficiency are more likely to report suboptimal SDM [6].

Shared decision-making in RA care is a priority among policy makers, clinicians and patients both nationally and internationally [7-10]. The demands on patients to have a basic level of knowledge of RA, the therapeutic options, and details of safety and efficacy of treatments when making a decision with their clinicians can be overwhelming, especially for those with barriers to communication like literacy or language. Competing tasks place clinicians at a disadvantage as they may not find time to inform patients of treatment options, discuss benefits and harms, and engage patients in shared decision-making.

Taking into account these increasing demands on patients and clinicians, evidence of suboptimal SDM, and disparities in outcomes, we set out to design a decision aid for use in the clinical encounter to facilitate conversation between patients and clinicians around issues related to RA medications. Here we describe and discuss lessons learned in the development of the decision aid.

We based the RA decision aid development on a process used to design a tool for patients with diabetes [11] which has been shown to enhance knowledge and improve patient involvement in decisions [12]. Breslin et al. noted that the shared decision-making model for patients with chronic disease must begin with partnership building, sharing of information, shared deliberation and, ultimately, joint decision making. They observed that patients make decisions about medications *after* leaving the clinician’s office, without the opportunity to ask questions, state their preferences or air concerns about medications. The motivation behind the diabetes decision aid design was to encourage patients to share concerns, raise issues, and ask questions *during the encounter* such that patient and clinician could arrive at a decision together. In this manner, clinicians could gain a shared understanding of the patient’s preferences, values, and context, and patients could make decisions about starting and using medicines informed by the clinician’s experience and expertise. Therefore, the goal of this project was to use a collaborative user-centered design process to create a tool, the RA decision aid, to facilitate patient engagement when choosing a new DMARD. Given the disparities in RA outcomes [2-4] and reports of poor shared decision-making communication among vulnerable populations, we chose to develop our tools for those with limited health literacy and include non-English languages of Spanish and Chinese. The concept of health literacy was addressed in the RA decision aid by following guidelines for developing low literacy materials (e.g., use of icons, plain language, more white space) as well as including patients of all literacy levels in the development process, and creating a tool that focused less on reading and more on conversation and verbal exchange of information. We adopted a collaborative approach, sometimes referred as participatory action research [13], to develop the decision aid. This process involves patients and clinicians – the users -- at every step of the process, as well as experts in the development of decision aids, health literacy, and designers to translate the materials into a tool that users would want to use.

## Methods

### Overview

Given that the majority of patients newly diagnosed with RA are started on methotrexate [14,15], we created our tool for the clinical context of an established RA patient with moderate to high disease activity who, at a minimum, has failed a trial of methotrexate. A 2013 study reported on the non-inferiority of triple combination synthetic DMARD therapy compared to adding a biologic [15] to methotrexate such that there is no one algorithm to direct medication choice in RA. At the time of the decision aid development, twelve DMARDs (4 synthetic and 8 biologic DMARDs) had been approved by the FDA and were commonly used in practice. While there are more than 12 FDA-approved DMARDs for RA, several are very rarely used in clinical practice.

**Figure 1:**
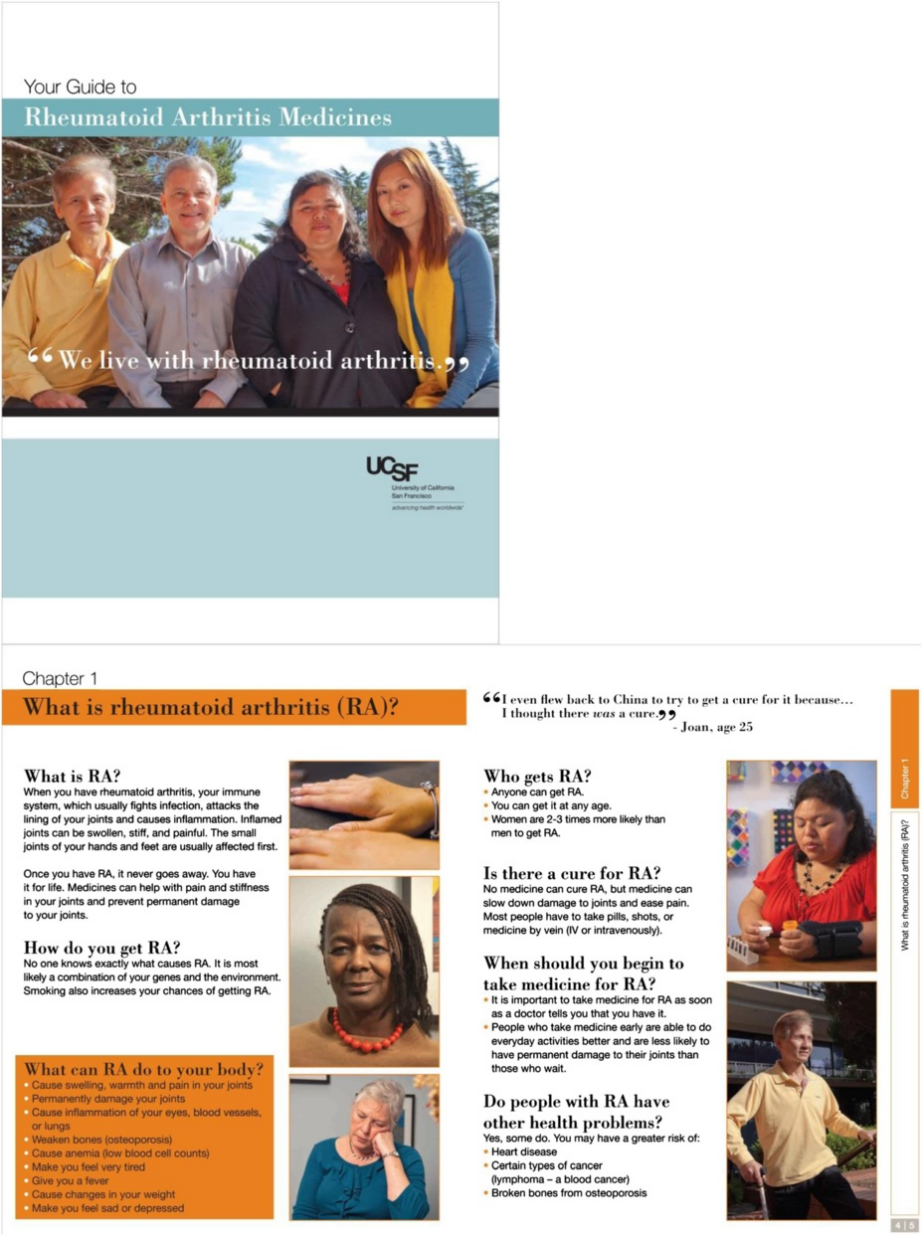
**Cover of the patient education material, “your guide to rheumatoid arthritis medications”, and two-page spread of chapter 1: “What is rheumatoid arthritis?”.**

The research protocol for this study was approved by the UCSF Committee on Human Research (IRB Number: 10-02339). All patient participants gave their written informed consent to be part of the study. All individuals whose images appear in the guide (Figure 1) signed written consent for permission to use their images for the purposes of the guide (research and education).

### The team

Using the design of the diabetes issue cards as a guide, we convened a diverse group of individuals with expertise in RA (clinicians and patients), decision aid research (VMM), design (TM, MB, DR), health literacy (JA, DS), clinical and qualitative research methods (LT, EY, CJK). We adopted the design approach described by Breslin et al. and encouraged all members of the team to bring methodologies and ideas from their respective fields to the table. This approach, with foundations in the design discipline, draws upon elements of field ethnography (which involves direct observation in clinic about how patients and clinicians discuss treatments) as well as participatory action research where both patients and clinicians contribute to the research by allowing participant observers (or third party researchers) to observe actual clinic visits. In addition, patients contributed by informing the researchers about content of the decision aid and evaluating prototypes both on an individual basis in cognitive interviews and as a larger group in a patient advisory board. Results from the cognitive interviews fed back directly into decisions regarding design, number of issue cards, and as a factor in how many cards were ultimately included in the final deck. Content from the interviews was discussed in team meetings and phone calls with consultants and designers.

Patients participated in two ways. The first was to invite patients from several previously conducted focus groups (one each in English, Spanish or Cantonese led by a bilingual, trained focus group moderator using a common interview guide that had been translated) to sit on a patient advisory board which met approximately every two months during the 18-month-long development process. The purpose of the initial focus groups was to gather data to inform the content and delivery of an adapted medication summary guide for RA medications as well as to explore patients’ perspectives on participation in decision making with their clinicians. The Grounded Theory Method [16] using constant comparison to determine similarity and differences within and between groups was used to analyze the transcripts [17]. We recruited heavily from the county hospital clinic for the focus groups, of whom over half had high school education or less. We then selected members for the board who had a range of literacy and education levels and continually emphasized the low literacy principles for the tool development. Of 9 board members, one-third had limited health literacy. The common language was English; however, several members were bilingual (either in Spanish or Cantonese). Cantonese, not Mandarin, is the predominant language spoken by Chinese speakers in San Francisco. Patients also participated during clinic visits at one of two clinics, the university-hospital based arthritis clinic or the county-hospital based RA clinic, when the prototypes were being tested. Clinical rheumatologists provided input as they tested the prototypes in clinic and offered feedback in research meetings.

### The development process

In addition to using the diabetes issue cards as a template, we followed six steps for the development of low literacy materials outlined by Seligman et al. [18] where the authors describe a process whose main purpose is to create materials which will increase knowledge as well as activate low literacy patients toward healthier behaviors. The six steps are presented in Table 1. Seligman et al. underscore the importance of mapping concepts onto a behavioral theory, such as the social cognitive theory, which suggests that materials should improve knowledge, positively influence outcome expectations, emphasize facilitators of behavior change, address barriers to behavior change, and facilitate the creation of goals [19].

**Table 1:** **Six steps for developing low literacy educational materials**

**Key concept**	**Study phase**
1. Convene a working team and solicit stakeholder input early	Phase 1 – Focus groups
	English-speaking RA patients
	Spanish-speaking RA patients
	Cantonese-speaking RA patients
	Rheumatologists
2. Identify key concepts to be communicated	Focus groups provide feedback on main areas of content:
	• Broad category of medicines for RA
	• Benefits of DMARDs across mono- and combination therapies
	• Talking with the doctor about RA drugs
	• Learning about risks
	• Ways to reduce risk
	• Cost
3. Map concepts to a behavioral theory, such as social cognitive theory and construct a brief intervention to support the use of written materials	Phase 1 -social cognitive theory suggests that materials should:
	• improve knowledge
	• positively influence outcome expectations
	• emphasize facilitators of behavior change
	• address impediments to behavior change
	• facilitate the creation of goals
4. Carefully design materials using low-literacy principles	Phase 2: adopt AHRQ summary guide for low literate, limited English language proficient patients
	• Use illustrations
	• ≥ 14 pt font
	• Adequate white space
5. Refine materials using input from the target population	Phase 2
	• In-depth, semi-structured interviews
	• Review adapted guides with patient advisory board
6. Assess success of efforts in target audience and learn from failures	Phase 3 – pilot test of decision aid, evaluate acceptability and outcomes of knowledge and decisional conflict

**Figure 2:**
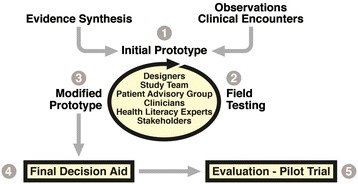
**Iterative process of the RA choice decision aid development.** Figure adapted from LeBlanc et al. [26] with permission.

The funder of this project, the Agency for Healthcare Research and Quality (AHRQ), published a summary of their comparative effectiveness review of RA medications for patients and clinicians [20,21]. Results from this review were used as the basis of the literature review for the decision aid. Additional systematic reviews of DMARDs and classes of DMARDs [22-25] were used to supplement the AHRQ review. In addition to gathering evidence-based reviews on DMARD safety, tolerability and comparative effectiveness, we also collected data on how medication discussions were taking place in clinics, as well as eliciting patient concerns about starting a new medication in focus groups, cognitive interviews, and from the advisory board. The process of combining data from the evidence synthesis and direct observations with feedback from various stakeholders is illustrated in Figure 2.

The development team reviewed and discussed issues that emerged from the literature review, clinical observations, and patient comments, and began to create prototypes of the cards. Each card was devoted to a particular issue, such as “side effects” or “cost” as based on the diabetes card design. The number, content, and design of the cards varied throughout their development. Clinicians were asked to use prototypes in clinical encounters where a third party (GEY) observed and recorded how the tool was used, the content of conversations around medications, and the level of patient involvement in the encounter. Prototypes were evaluated in recorded one-on-one interviews with a member of the research staff (LT, JB) and RA patients, as well as non-RA patients (in role play) to gather insights into the content and design of the tool and its ability to generate a conversation. Mock clinic visits and actual clinic visits using the prototypes were then observed by the designer (TM) and other team members (LT, GEY) after which they provided feedback on the nature of the conversations. Health literacy experts also reviewed the prototypes.

In discussions with the patient advisory board, we sought to identify any issues that the cards did not cover and therefore expand the repertoire of issues. Most importantly, we examined whether the cards accomplished the goal of creating a conversation between patient and clinician. Iterations of the tool continued until the main patient issues and concerns were covered and the cards were consistently generating an exchange between clinician and patient about RA medications. This entire process took approximately eighteen months.

### Role of the funding source

AHRQ funded this project and played no other role in this work.

## Results

### Developing the decision aid

#### Content

The content was derived from the AHRQ and other evidence-based reviews as depicted in Figure 2. The DMARDs presented in the original AHRQ summary guide included anakinra which upon discussions with local rheumatologists was deemed to be used exceedingly rarely in the treatment of adult RA. Therefore, anakinra was not included. A total of twelve DMARDs were included: synthetic DMARDs - hydroxychloroquine, leflunomide, methotrexate, sulfasalazine; biologic DMARDs - abatacept, adalimumab, certolizumab, etanercept, golimumab, infliximab, rituximab, tocilizumab. The newest FDA-approved DMARD for RA, tofacitinib [27], received its approval after development was completed and is not included.

Route of administration, frequency of dosing, and side effects vary among DMARDs and play a role in patient preferences for choosing an RA medication [28-33]. Other important patient issues that emerged in focus groups, advisory board meetings and in clinical encounters included cost, benefits, and time until clinical effects are experienced. Clinician concerns included special considerations such as pregnancy (some DMARDs are teratogenic), latent or active tuberculosis (all biologic therapies increase the risk for opportunistic infections including TB), and alcohol consumption (methotrexate and leflunomide can be hepatotoxic; heavy alcohol consumption is contraindicated). Clinicians aware of the benefits of combination therapy using two or more DMARDs to achieve greater efficacy were interested in a combination DMARD issue card as well as one which would address goals of therapy for RA.

#### Form and information design

The team decided *a priori* to use the issue card format developed by Breslin and colleagues at the Mayo Clinic. Therefore, our prototypes varied over time in the number of issue cards in each “deck”, and the design and content of each card. At three distinct time-points we had a collection of cards which varied by number and appearance, as discussed below.

#### First prototype deck

Our initial set of cards consisted of six issues: medication routine, cost, results (time to onset), side effects, infection risks, and special considerations. Ten medications (methotrexate and rituximab were not initially included) were organized with pills on one card and then IV/subcutaneous injectables on another. We then combined all medications onto one card in a consistent way across all issues grouping them by mode of administration: oral, subcutaneous (“shots under the skin”) and intravenous delivery (“given in the vein”). Following low literacy principles to use more illustrations, the research team explored the use of images: photographs of pills, a person giving himself an injection, and an arm with an IV as well as cartoons or icons.

We elicited feedback from a group of rheumatologists and from the patient advisory board on this first iteration. Physicians overall were concerned that the cards “may open a can of worms” and prolong the length of the clinical encounter, however they felt certain cards (like the “routine” card) were very important. Specific clinician feedback included suggestions to add icons for pills/IV/subcutaneous injections; separate common from rare and mild from severe side effects; and add a “benefits” card to show efficacy. Clinicians emphasized that they often have their own order in which they present medication options (e.g., 1^st^ line, second line, third, etc.) and were concerned that patients may interpret the order in which the medications are listed on the cards as an order of preference. Physicians felt that the cost issue was very individualized depending on patient insurance and that it may be more important to first make the decision regarding which medication and *then* deal with cost. Also, they were concerned that patients may perceive this card in a negative way, as in: “why should we care about costs?” Clinician concerns around cost were misaligned with those of patients who expressed interest and concern about medication cost in focus groups and during prototype reviews. One Cantonese-speaking patient upon seeing the actual costs of a biologic commented: “If you want me to pay, I prefer to die. Where can I get the money?”

Patient advisory board members piloted the cards using role play with one another and two researchers (GEY, LT). During this exercise, several patients took on the role of a patient and the other a doctor while a third party observed the conversation. Elicitation of values and concerns were observed during the use of the cards, cost was discussed frequently as was mode of administration and onset of action. No issue cards were felt to be missing from the discussion though one patient wanted to see prednisone (which is not traditionally considered a DMARD) on the cards.

**Figure 3:**
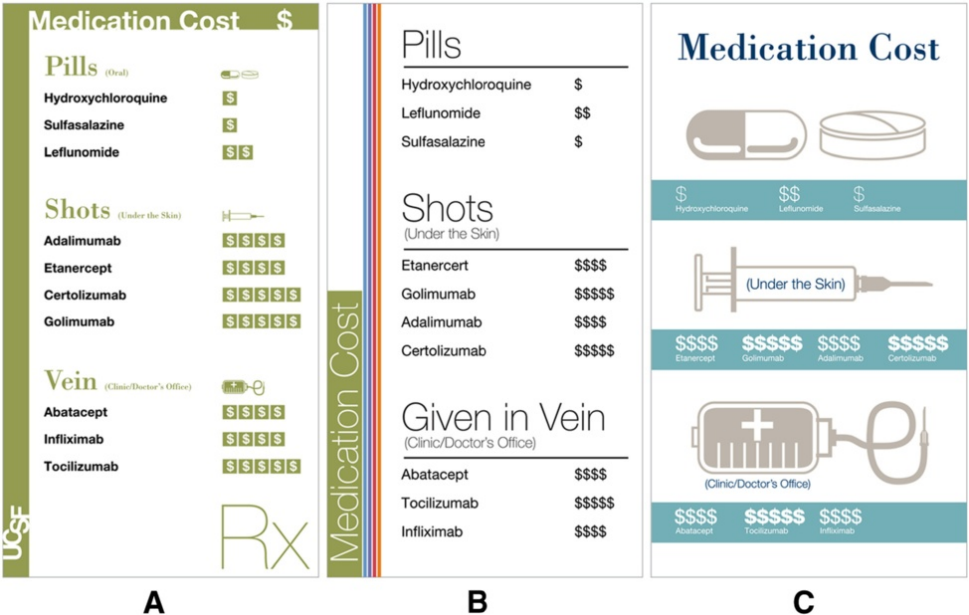
**First iteration of the issue cards.** Note the variation in the size of text, fonts, use of icons, and organization of medication names. Six issue cards were included in this “deck”: routine, cost (as seen above), results (onset of action), side effects, infection risk and special issues. **(A)** Design 1, **(B)** Design 2, **(C)** Design 3.

The designers then piloted three different visual designs (Figure 3).

#### Second prototype – deck #2

**Figure 4:**
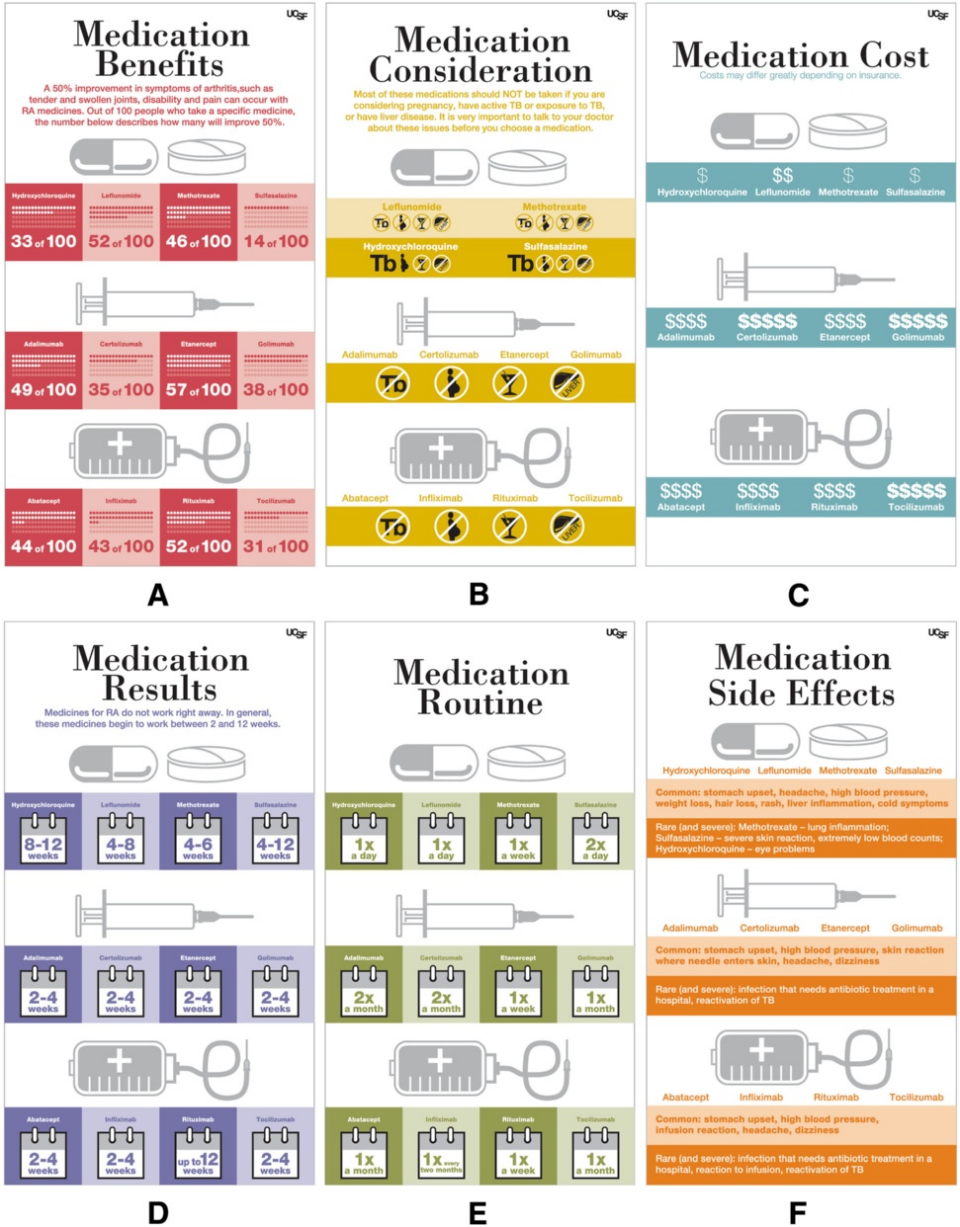
**Second iteration of issue cards.** This deck included one new issue card on benefits, and collapsed the infection risk card into the side effect card for a total of six cards: **(A)** benefits, **(B)** considerations (special issues), **(C)** cost, **(D)** results, **(E)** routine, **(F)** side effects.

The research team and designers, with feedback from the patient advisory board, selected their preferred font, colors, icons, and layout for a consistent visual presentation in this next iteration (Figure 4). In addition to the design revision, a “benefits” or efficacy card was added in response to feedback, and infection risk was incorporated into the side effects card. For the benefits card, the research team chose to use pictographs to illustrate efficacy given a growing body of evidence showing that pictographs are more quickly and better understood than other graphical formats [34]. The benefits card illustrated the number of people out of 100 who achieve an American College of Rheumatology (ACR) 50 response which reflects a 50% improvement in tender and swollen joints and 3 of 5 additional criteria [35] to each of the listed medications. This is a standard measure of efficacy included in RA clinical trials and data for this domain was extracted from the literature review.

**Figure 5:**
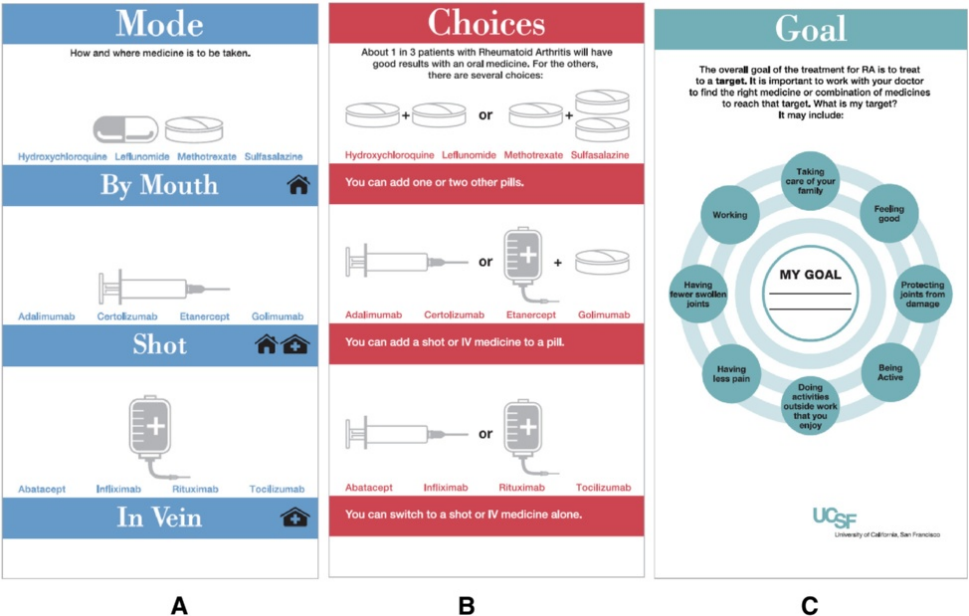
**Three issue cards added to deck #2 based on clinician feedback: Mode of administration, combination therapy, and goals of therapy. A**. Mode of administration prototype. **B**. Prototype of combination therapy issue card. **C**. Prototype of goals issue card.

Ongoing clinician feedback led to prototypes of three additional cards: mode of administration, combination therapy, and goals. The “mode” card (Figure 5a) illustrated route of administration using icons of a pill, a pre-filled syringe and an infusion bag with tubing and needle as well as icons of a home and clinic/hospital to depict the setting. The combination therapy card (Figure 5b) came out of discussions with clinicians who are aware of the evidence that combination therapy of a synthetic DMARD such as methotrexate with a biologic has superior efficacy than monotherapy with either agent [36]. This is also true of combination therapy with synthetic DMARDs (e.g., triple therapy with methotrexate, hydroxychloroquine and sulfasalazine) [37]. Clinicians and the research team also felt that prior to initiating a discussion of the issue cards with a patient, clinician and patient goals for therapy be established. Goal concordance between patient and provider has been shown to lead to better outcomes in other chronic diseases [38,39]. This prompted the prototype of a “goals” card (Figure 5c).

#### Final decision aid tool – deck #3

**Figure 6:**
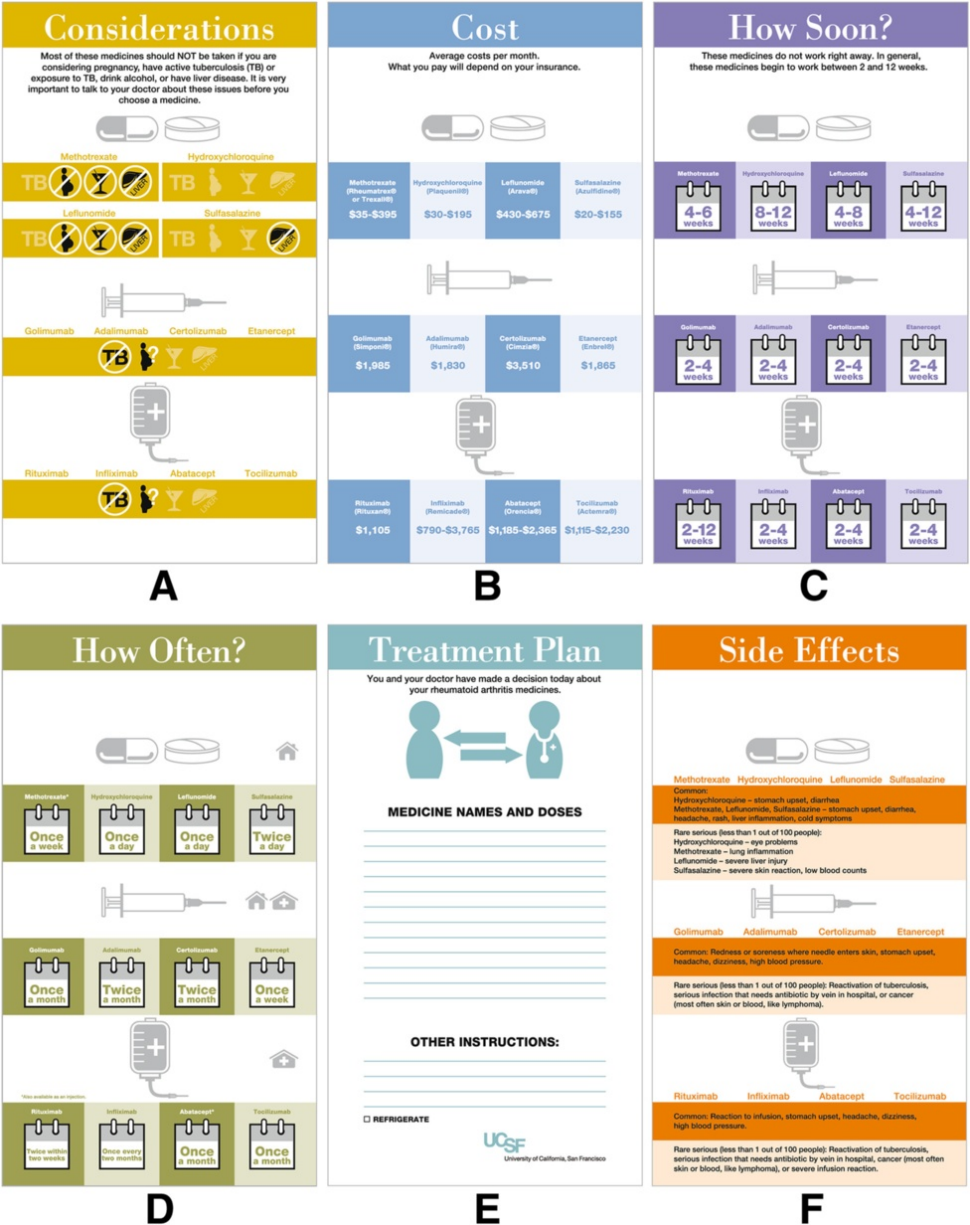
**Final set of five issue cards and a cover page (F) for a tri**-**fold copy of the cards given to patients at the end of their clinic visit. (A)** Considerations, **(B)** Cost, **(C)**, How Soon?, **(D)** How Often?, **(E)** cover page, **(F)** Side Effects.

**Figure 7:**
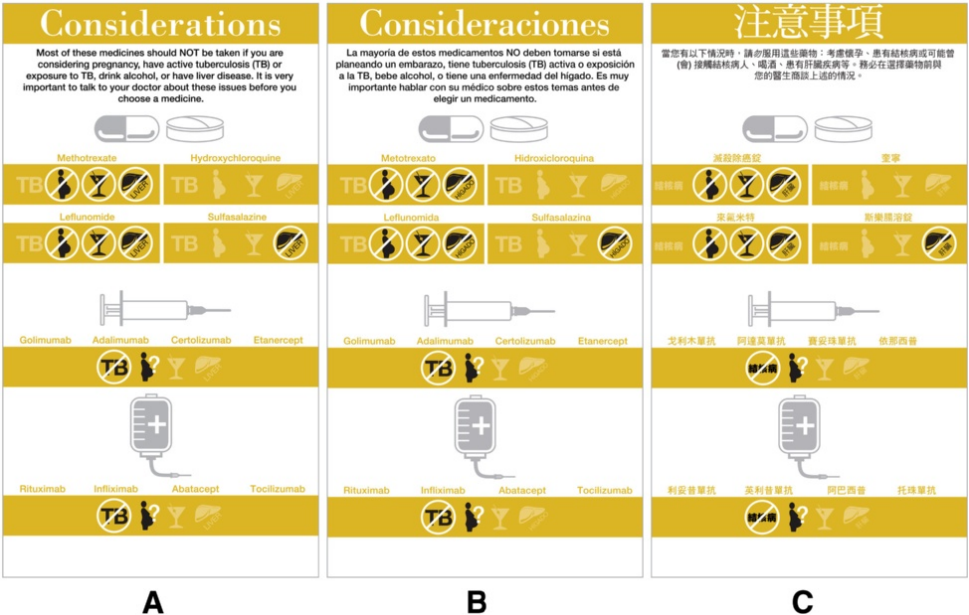
**Illustration of the special considerations issue card in each of the three languages:** **(A)** English, **(B)** Spanish and **(C)** Chinese.

Several changes were made to this final deck in response to feedback from multiple stakeholders. Clinicians were extremely concerned about the amount of time it would take to review all eight issue cards (including the goals and combination therapy card) however they did note that the cards pushed them to consider patient preference as well as their own which they acknowledged promoted patient-centered care (their default was to tell the patient which drug they preferred, as opposed to eliciting patient preferences; the cards helped initiate that conversation). Patients reported some difficulty reading lighter color text on top of color, but liked the icons and calendar depictions. In cognitive interviews, it became clear that the goals card generated tangential conversations which distracted patients and providers from the task at hand. The combination card did not add to the conversations and it was deemed by our decision aid expert to be a “doctor” card in that it was information the doctor could easily convey in a sentence and was not an issue of importance to the patient. With respect to time, visits during the initial prototype testing varied from 30 to 60 minutes. We attributed some of this to a lengthy introduction of the tool to patients as well as a greater number of issue cards in the deck (Deck #2 - 8 cards). Three cards were then eliminated from the deck (goals, benefits, combination therapy) and a more stream-lined approach to using the cards in the clinic was developed. With these adjustments, visit length on average was 33 minutes (range 12-57 minutes) and often did not take longer than a visit without the tool. One user reported an increase visit time of 2 minutes with the tool. Clinicians were asked to start the conversation about choosing a medication with three main issue cards (how often, how soon and cost), and if patients asked questions about side effects or raised concerns related to special considerations, those cards would be pulled into the conversation. Thus the team arrived at a final set of five issue cards to be used in the clinical encounter (Figure 6). The final tool was translated and back-translated [40] into Spanish and Chinese by a professional translation agency (Figure 7).

## Discussion and conclusion

### Discussion

The development of a low literacy medication decision aid (RA Choice) in three languages was feasible using a collaborative process with patient and clinician input at every step. There is a clear need for increased support and structure to promote and facilitate shared decision-making in RA, especially among more vulnerable populations with poorer health outcomes and barriers to communication like literacy and language. Decision aids are one way to promote patient-centered care [41]. There are several decision aids for patients with RA [42-44], however none have been developed for patients with limited health literacy or limited English language proficiency for use in the clinic encounter.

During our development process, certain decisions had to be made to respect clinician concerns about consultation time, inability to have comprehensive information on the cards and meet standards for a low literacy tool, and be cognizant of not overwhelming patients with too much information. We based our tool development on a process described for a diabetes decision aid, the overarching goal of which was to create a conversation between patients and clinicians. Both diabetes and RA are chronic conditions with multiple options for therapy requiring a high degree of self-management. Given the parallels between the two conditions and choices for therapy, the diabetes issue card design was an excellent template for the RA decision aid.

Our process was not without limitations. While we created the decision aid and translated (and back-translated) it into Spanish and Chinese, we found it difficult to fully involve Cantonese-speaking patients and research staff in the process. Due to time constraints we were unable to thoroughly pilot the tools with Cantonese speakers, interpreters, and clinicians in the clinics. There are both language and cultural adaptions which may enhance the use and efficacy of RA Choice given the opportunity to observe a broader number of encounters and this is a goal for future projects. More specifically, lessons learned for future work include a focus on a single language during development, more in depth cognitive interviews with Cantonese speakers, and to determine the best way, without using names or characters, to share information on medications (of note, only one of 7 Cantonese focus group participants could recognize the written name of their RA medications and relied on color and size of pill). In addition, background research on shared decision making preferences in these populations is needed. We chose to create a print tool (as opposed to web-based) which may limit its usability in clinics which are fully electronic, as well as limit, if effective, our ability to distribute it and keep it up-to-date without having to recall distributed cards. It may also make it more difficult to keep the tool updated with new medications or comparative effectiveness data. A web-based version of the tool may be more appropriate once exam rooms are better equipped for full participation of patients and providers and the investment justified by evidence of efficacy of the cards. It is also a challenge to create a design that appeals to all users, therefore an electronic tool tailored to each individual may have greater reach as well as be easier to update. The state of the comparative effectiveness literature made it impossible to accurately depict benefits (lack of head to head trials for all biologics) and the research team ultimately concluded that using a measure such as the ACR 50 misled patients (i.e., depicting one medicine as more effective, leading to more patients achieving an ACR 50, when head-to-head trials are either not available or suggest no difference). This led to a decision not to include an issue card on efficacy. The complete experience of the decision aid tool in the clinical encounter relies on the communication skills of the clinician to engage the patient in conversation and of the patient to engage in conversation and for both to use the tools as intended. In contrast to the development of the diabetes decision aid, we were not able to pilot test the cards in actual clinical encounters in high numbers consistently across the tool development (more so in the beginning stages) due to the fact that clinics would be sites for a pilot trial.

The tool, in and of itself, does not meet all International Patient Decision Aid Standards (IPDAS) Collaboration criteria, which were designed for standalone tools directed at patients. However, cumulative, including through this publication, the tool satisfies criteria for balanced evidence-based and up-to-date information, depiction of relevant issues using state-of-the-art approaches, and designed for purpose and setting [45]. The categories of the IPDAS criteria that were not met include: a description of the positive features (as discussed above a benefits issue card is not included in the deck), presentation of probabilities of outcomes (to do so would have added significant complexity to the design of the tool which in turn would create excess burden on patients with limited health literacy). In terms of values clarification, this takes place upon the introduction of the issue cards by the clinician when he or she asks the patient to “pick which issue you would like to discuss first” about a new medication. The act of allowing the patient to hold and select an issue card is a concrete way to allow the patient to clarify and express his or her values. By providing a copy of the decision aid in the form of a trifold pamphlet as well as the medication summary guide to take home, patients are also encouraged to share with family or friends what may matter most to them in choosing a medication – be it cost, mode of administration, side effects, etc.

### Conclusion

The development of a low literacy decision aid for RA medications in three languages was feasible using a collaborative process with patient, clinician and design input. Our decision aid, which is intended to be used in the context of an RA patient with moderate to high disease activity despite being on at least one DMARD, promoted a conversation and exchange of experience on the part of the clinician and values and preferences on the part of the patient. It is our expectation that the use of RA Choice in the clinical encounter will foster more patient-centered care and enhance shared decision-making for all RA patients.

### Practice implications

A clinical trial to test the final prototype of RA Choice is needed to determine its impact on patients and clinicians, its feasibility for use in usual care settings, and its impact on process and outcomes of care.

Role of funding: Funding for this project was from the Agency for Healthcare Research and Quality (R18 HS019209-01, PI Yelin). AHRQ had no role in study design; in collection, analysis and interpretation of data; in the writing of this manuscript; or in the decision to submit this paper for publication. Dr. Yelin, Ms. Trupin and Ms. Evans-Young also received funding from the NIH (NIAMS P60 AR053308).
